# Suspended sediment load prediction using long short-term memory neural network

**DOI:** 10.1038/s41598-021-87415-4

**Published:** 2021-04-09

**Authors:** Nouar AlDahoul, Yusuf Essam, Pavitra Kumar, Ali Najah Ahmed, Mohsen Sherif, Ahmed Sefelnasr, Ahmed Elshafie

**Affiliations:** 1 Faculty of Engineering, Multimedia University, 63100 Cyberjaya, Malaysia; 2 Institute of Energy Infrastructure (IEI), Department of Civil Engineering, College of Engineering, Universiti Tenaga Nasional (UNITEN), 43000 Selangor, Malaysia; 3 Department of Civil Engineering, Faculty of Engineering, University of Malaya (U.M.), 50603 Kuala Lumpur, Malaysia; 4 National Water and Energy Center, United Arab Emirates University, P.O.Box: 15551, Al Ain, United Arab Emirates; 5 Civil and Environmental Engineering Department, College of Engineering, United Arab Emirates University, P.O.Box:15551, Al Ain, United Arab Emirates

**Keywords:** Hydrology, Engineering

## Abstract

Rivers carry suspended sediments along with their flow. These sediments deposit at different places depending on the discharge and course of the river. However, the deposition of these sediments impacts environmental health, agricultural activities, and portable water sources. Deposition of suspended sediments reduces the flow area, thus affecting the movement of aquatic lives and ultimately leading to the change of river course. Thus, the data of suspended sediments and their variation is crucial information for various authorities. Various authorities require the forecasted data of suspended sediments in the river to operate various hydraulic structures properly. Usually, the prediction of suspended sediment concentration (SSC) is challenging due to various factors, including site-related data, site-related modelling, lack of multiple observed factors used for prediction, and pattern complexity.Therefore, to address previous problems, this study proposes a Long Short Term Memory model to predict suspended sediments in Malaysia's Johor River utilizing only one observed factor, including discharge data. The data was collected for the period of 1988–1998. Four different models were tested, in this study, for the prediction of suspended sediments, which are: ElasticNet Linear Regression (L.R.), Multi-Layer Perceptron (MLP) neural network, Extreme Gradient Boosting, and Long Short-Term Memory. Predictions were analysed based on four different scenarios such as daily, weekly, 10-daily, and monthly. Performance evaluation stated that Long Short-Term Memory outperformed other models with the regression values of 92.01%, 96.56%, 96.71%, and 99.45% daily, weekly, 10-days, and monthly scenarios, respectively.

## Introduction

Suspended sediment is generally referred to as sediment within a water body such as a river, which is transported by fluid and is fine to the point that turbulent eddies are able to outweigh the settling of the sediment particles within the water body, causing them to be suspended^1^. The deposition of sediment in rivers is a well-known and costly issue that affects environmental health, agricultural activities, and potable water sources. This is due to its detrimental impacts on water quality, which causes the pollution of water bodies, particularly rivers^2^. Under certain conditions, suspended sediments also interfere with a river’s normal hydrological system^3^. When the river channel's velocity and momentum reduces, the suspended sediments may start to accumulate at the river channel’s bottom, causing the elevation of the river channel’s bottom, hence reducing the cross-sectional area of the river channel and choking the river’s hydrological system^4^. This, in turn, reduces the habitat of aquatic creatures residing in rivers.

Due to the reasons above, the investigation and accurate prediction of suspended sediment load (SSL) is crucial for the long-term preservation and conservation of river health, as well as for human activities and necessities, namely agriculture, potable water supply, while also handling issues relating to the design, planning, and operations, of hydraulic structures namely dams and reservoirs; and comprehensive environmental impact assessments^2,5^.

In this application of SSC prediction, various challenging issues are available. Firstly, SSC prediction differs from one site to another, and thus it should be modelled for every river utilizing data collected in this specific site. Secondly, our data used for training the model have only one factor: daily discharge data used to predict the daily suspended sediment. Thirdly, daily sediment data have complex nature, which leads to less forecasting accuracy. Therefore, to address previous challenges, this study proposes a Long Short Term Memory, a well-known model that can detect complex patterns in data. This study aims to predict the suspended sediments in the Johor river in Malaysia.

## Literature review

Machine learning (ML) algorithms have been widely used in solving many complex problems. For example, a feedforward neural network was developed to predict the groundwater level of the lagoon of Venice^6^. Radial basis neural network also proposed to predict the water level of Venice lagoon^7^. There have been many studies that investigate the prediction of SSL using various machine learning (ML) algorithms^8–10^. The first type of ML model reviewed is standalone ML models, which have been demonstrated to be capable of predicting SSL. Choubin et al*.*^11^ conducted research on the classification and regression tree (CART) algorithm's capability, also known as the decision tree (D.T.) algorithm, in modelling suspended sediments in the Haraz River, Iran. The performance of the CART-based model and the other standalone models based on the multilayer perceptron (MLP) neural network, support vector machines (SVM), and the adaptive neuro-fuzzy inference system (ANFIS) was compared, and it was concluded based on three performance measures that the CART model is superior compared to the other standalone ML models in predicting SSL.

Talebi et al*.*^12^ performed a study that investigated the usage of the CART algorithm, the M5 decision tree (M5T) algorithm, ANN, and the conventional sediment rating curves (SRC), in predicting SSL in the Hyderabad drainage basin in Iran. The results showed that the CART and M5T algorithms outperformed the other models. It was found that the conventional SRC method had high accuracy in predicting the daily discharge of sediments of less than 100 tons per day, while the prediction of high sediment discharge was more accurate by the ML models compared to the conventional SRC method.

Nivesh and Kumar^5^ used two standalone ML models: ANFIS and multiple linear regression (MLR), along with the conventional SRC model, to predict SSL in the Vamsadhara River basin India. Three different input scenarios were trained with these models, with the study concluding the ANFIS-based model is the better performer in predicting SSL compared to MLR and SRC.

Nivesh and Kumar^13^ also performed a study that compared the performance of another two standalone ML models, namely artificial neural network (ANN) and MLR, in predicting SSL in the Vamsadhara River basin in India. Based on three different performance indicators, it was found that ANN is better and more efficient in predicting SSL compared to MLR.

Taşar et al*.*^14^ utilized three ML algorithms, namely ANN, M5T, MLR, and the conventional SRC method, to predict suspended sediment in Iowa, United States. Based on the comparison of three performance indicators, it was found that ANN is superior in forecasting suspended sediment compared to the M5T, MLR, and SRC methods.

Although standalone ML algorithms are capable of predicting SSL, they inhibit several limitations that should be noted. Generally, it can be understood that standalone ML models are not as accurate or robust as hybrid ML models^15^. Hybrid ML models are often more reliable because they gain advantages from their constituent algorithms, whereas standalone ML models do not gain this benefit^15^. In the context of SSL prediction, the superiority of hybrid ML models over standalone ML models can be observed in previous studies^2,15,16^.

Qian et al*.*^17^ stated that standalone ML models are also less capable of coupling and processing nonlinear problems compared to hybrid ML models. Several other disadvantages, depending on the standalone ML algorithm used, are overfitting, lack of memory, parameter uncertainty, cognitive uncertainties, and local minimization drawback, the requirement to comply with data assumptions, ability to only provide linear solutions, assumption of independence between features, and requirement of large data samples to achieve good performance^15,18^.

Previous literature on hybrid ML models capable of predicting SSL has also been reviewed. To estimate SSL in the Mahabad River, Iran, Mohammadi et al*.*^2^ hybridized an MLP with particle swarm optimization (PSO). This hybrid algorithm was then integrated with the differential evolution (D.E.) algorithm. The resultant algorithm was called MLP-PSODE. This algorithm's performance was compared with another hybrid algorithm that is MLP-PSO, which is similar to MLP-PSODE but without integration of the D.E. algorithm, and several other standalone algorithms, namely MLP, SVM, and radial basis function (RBF). The study found that MLP-PSODE is better compared to MLP-PSO and the standalone models in the case of estimating SSL, as it is more accurate in extreme value estimation.

Banadkooki et al*.*^19^ performed research investigating SSL estimation in the Goorganrood basin, Iran using an ANN model hybridized with the ant lion optimization algorithm (ALO). Two other hybrid ANN models were also studied: ANN-PSO and ANN-BA, which are ANNs hybridized with the particle swarm optimization (PSO) and the bat algorithm (B.A.). Several input scenarios were tested to examine the capabilities of the hybrid ML models, with results showing that the ANN-ALO models had better accuracy than the ANN-PSO and ANN-BA in estimating SSL.

Ehteram et al*.*^20^ studied the usage of hybridized multilayer feedforward neural network (MFNN) and ANFIS in improving the prediction of suspended sediment in the Atrek River, Iran. Two MFNN models were hybridized with the weed algorithm (W.A.) and the bat algorithm (B.A.), producing models called MFNN-WA and MFNN-BA, respectively. Two ANFIS models were also hybridized with W.A. and B.A., producing models called ANFIS-WA and ANFIS-BA, respectively. The study concluded ANFIS-BA was the best in predicting SSL compared to the other hybrid ML models, based on five performance indicators.

Adnan et al*.*^4^ developed three models to predict SSL at Guangyuan and Beibei, China. The models are a dynamic evolving neural fuzzy inference system (DENFIS), a multivariate adaptive regression splines (MARS), and an ANFIS model hybridized with fuzzy c-mean clustering (ANFIS-FCM). Using selected standard performance indicators, it was concluded that the DENFIS model showed improved accuracy compared to the MARS and ANFIS-FCM models in predicting SSL. Zounemat^21^ performed a study on the San Joaquin River, United States, regarding the forecasting of suspended sediment concentration using two ANN models hybridized with the Levenberg–Marquardt (L.M.) algorithm and PSO, which are called ANN-LM and ANN-PSO, respectively. A standalone ANFIS model was also developed. The study finds that ANN-PSO and ANFIS were superior in predicting daily suspended sediment concentration values.

Hybrid ML models also have limitations that need to be considered in solving problems such as SSL prediction. Qian et al*.*^17^ stated that the training time of hybrid ML models is high, especially when dealing with complex problems. Hybrid ML models require many more input parameters to be considered during training compared to standalone ML models. This often restricts the development and usage of hybrid ML models^17^. In addition, complicated architecture and an unknown optimal number of clusters have also been reported as disadvantages of utilizing hybrid ML models^15^.

One type of ML algorithm, which has not been explored much in the context of SSL prediction, is the convolutional neural network (CNN). This neural network, which is a kind of deep learning algorithm, has shown plenty of promise in other fields based on previous literature reviews. Kabir et al*.*^22^ developed a CNN to predict flood depths in Carlisle, United Kingdom. The CNN model developed in this study was trained with outputs provided by a two-dimensional (2D) hydraulic model. The CNN model's performance was compared to that of a support vector regression (SVR) model. This study determined that the proposed CNN model was far superior to the SVR in predicting flood depths, as indicated by several adopted performance measures.

Haurum et al.^23^ investigated the usage of CNN in estimating the water levels in sewer pipes in Denmark. Models based on the decision tree algorithm were also trained and tested for performance comparison with the CNN model. The estimation problem in this study is treated as a classification and regression problem. This study demonstrates that the CNN models outperform the decision tree models, in the context of estimating water levels.

Huang et al*.*^24^ utilized a CNN trained using a robust loss function to forecast the river flow in four rivers in the United Kingdom. The performance of the CNN model trained using a robust loss function is compared with benchmark models based on several algorithms, namely autoregression (A.R.), radial basis function neural network (RBFNN), MLP, kernel ridge regression (KRR), and a generic CNN. This study shows that the CNN trained using a robust loss function produces the best forecasting performance.

Ni and Ma^25^ researched the applicability of implementing a model based on CNN to predict the generation of power from a marine wave energy converter (WEC) system through the utilization of a double buoy oscillating device (OBD). A multi-input approach was used to train and test the CNN. The study concludes that the proposed CNN model performs better than the ANN and regression models in the prediction of marine wave power generation.

Zhu et al.^26^ studied the utilization of CNN in developing a model to predict the generation of wind power. Wind power historical data obtained from a wind farm is fed to the CNN model as input to predict wind power generation 4 h ahead. This study, which is the first to use CNN to predict wind power generation, proves that CNN is indeed feasible for application in regression prediction in order to predict wind power generation.

There are many advantages in utilizing CNNs to solve problems in engineering-related and non-engineering-related fields. Among the primary benefits of using CNNs is that they are machines that learn end-to-end, with images of input mapped directly to the target bounding box coordinates or goal labels^27^. This direct mapping ability means that the design of suboptimal handcrafted features, also known as feature engineering (F.E.), is a time-consuming process and may cause image representation to be noisy with suboptimal discriminative power, which is no longer needed^27,28^. CNN's are also robust and rugged to challenging situations such as distortion in images, which are commonly caused by shape change due to camera lens, varying lighting conditions, partial occlusions presence, varying poses, and horizontal and vertical shifts^28,29^. In addition, with the same coefficients used throughout different locations within the space of the convolutional layer, the memory requirement is significantly reduced for CNNs^29^. The training time of CNNs is also reduced, as the number of parameters is substantially reduced, making training more manageable and better 29 while also making processing faster^30^.

## Problem statement

The usage of sub-optimal models or methods in measuring, calculating, and predicting SSL is costly in terms of time, funding, energy, and workforce^2^. The sediment rating curve (SRC), which utilizes a regression analysis to establish a relationship between sediments and river discharges, is a conventional and standard means of predicting SSL^12,14^. However, it has been found to be incapable of providing sufficiently accurate predictions, as the procedure of utilizing sediment loads versus stream discharge has been shown to be inaccurate^14^. Because of this, researchers have turned themselves in the direction of artificial intelligence (A.I.) and its subset, which is machine learning (ML). ML is able to identify trends and patterns with ease; operate automatically; continuously improve; and handle data of multi-dimensions and multi-variance, which makes it especially useful in utilizing large amounts of data to predict SSL.

Traditional ML methods that were previously mentioned in the literature review depended on feature engineering to select features manually before the prediction stage. If the features were not selected carefully, the prediction performance would degrade. Moreover, the selection of hyperparameters is critical and has an enormous impact on prediction performance. Additionally, conventional ML methods were found to degrade the performance of the patterns in the data are complex. More advanced automatic learning methods such as deep learning models were demonstrated to learn this type of complex pattern.

Deep learning models such as CNNs, which were demonstrated in SSL application, focused only on spatial features to extract features related to current input and ignore other features available in the previous time steps. Therefore, the recurrent neural network was the key solution to automatically extracting the temporal features and keeping track of all historical features to consider the relation between current and previous sediment and discharge samples. When the history of features or dependencies is long, traditional RNNs suffer from performance degradation due to gradient vanishing problems^30^.

## Objectives

In this research work, we utilized LSTM, which was found to solve the vanishing problem^31^ and improve performance by considering a large number of sediment and discharge values collected from previous days, weeks, 10-days, and months.

The purpose of this study is to explore the capability and demonstrate the effectiveness of a model based on long short-term memory (LSTM) neural networks in predicting suspended sediment load (SSL) in the Johor River basin, given a time series of historical data relating to suspended sediment and river streamflow. The observed and predicted SSL values are inspected comprehensively through statistical analyses. After predicting SSL, the performance of the LSTM model is examined and evaluated using several selected performance indicators to determine the efficacy of LSTM in the field of SSL prediction.

## Methodology

### Study area

Located in Southeast Asia, Malaysia is primarily made up of two land regions: Peninsular Malaysia and the Borneo Islands, which consists of the states Sabah and Sarawak. The air in Malaysia is generally moist and cloud-covered, as the sea surrounds the country. The country is also situated near the equator; hence it receives higher concentrations of sunlight, as rays from the Sun almost entirely strike throughout the year. The case study area, Johor, is situated in the southern parts of Peninsular Malaysia. Johor is officially segregated into a total of eight districts, with the capital of the state being Johor Bahru which is highly urbanized as it serves as a port of entry connecting countries Malaysia and Singapore while also acting as an international business hub. The district of Kota Tinggi, which has an area of 3644 km^2^ and is based approximately 42 km north-east of Johor Bahru, has rapidly developed as part of the growth corridor of Johor due to its close proximity to the Johor capital. Kota Tinggi, located at East Johor with 10 sub-districts, has the sea encompassing 65% of its border^32–34^.

**Figure 1:**
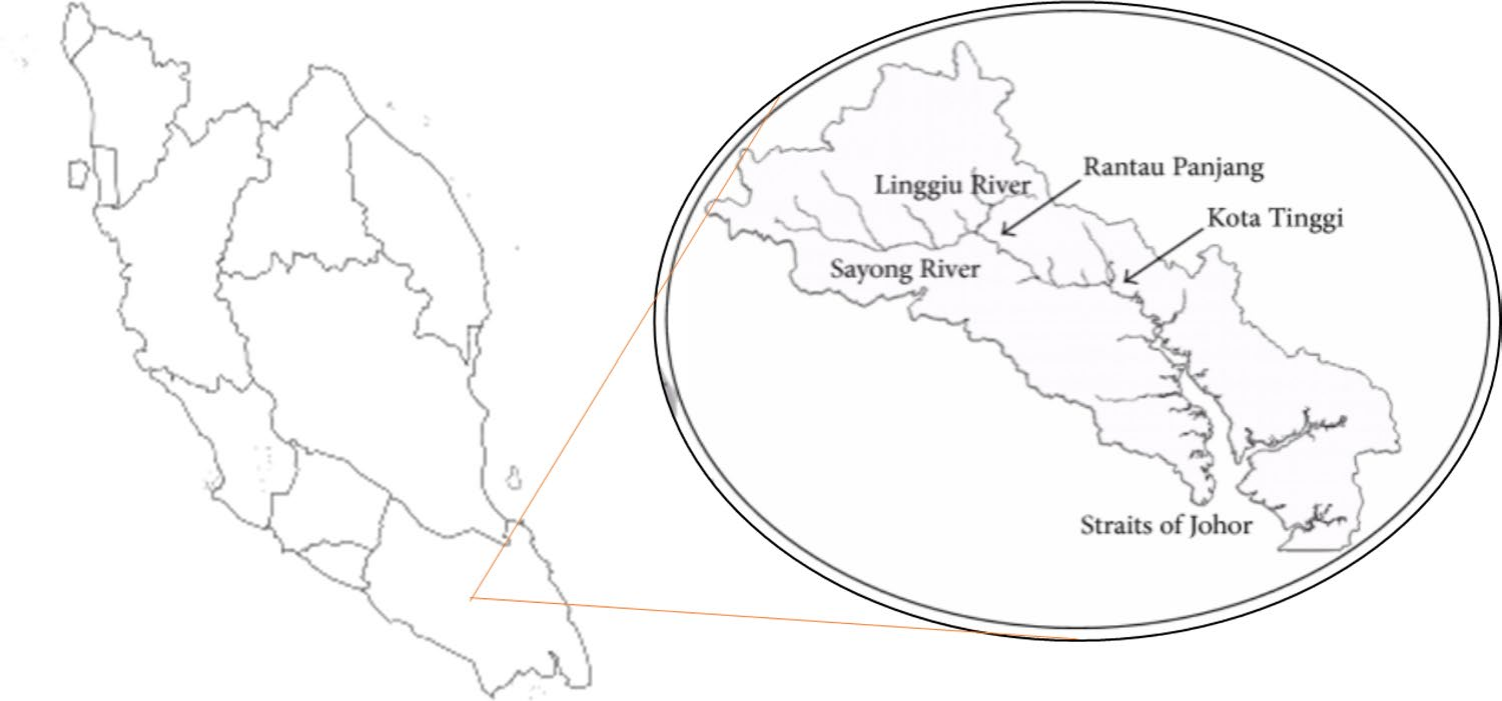
Illustration of the Johor River basin^35^.

**Figure 2:**
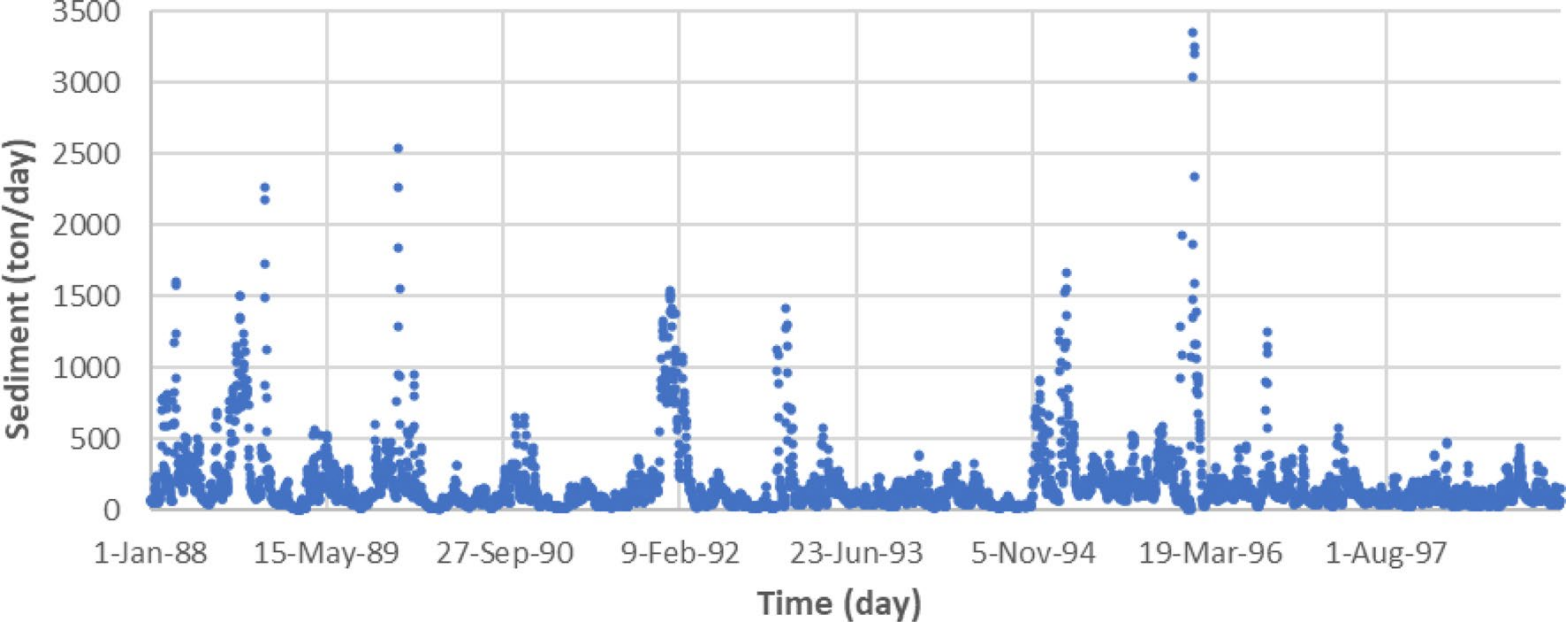
Sediment vs. time scatter plot for the Johor River basin from 1988 to 1998.

**Figure 3:**
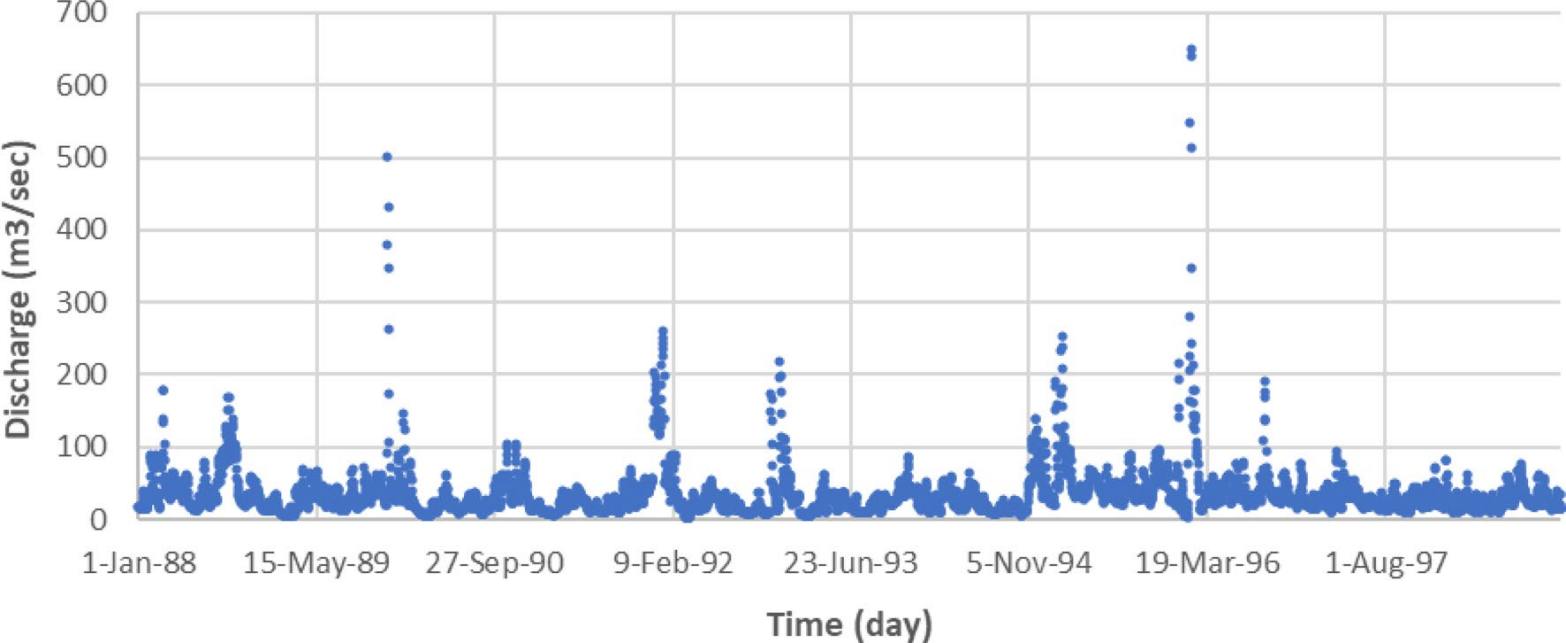
Discharge vs. time scatter plot for the Johor River basin from 1988 to 1998.

This study uses the Johor River basin as the case study area, as illustrated in Fig. 1. The Johor River basin comprises of approximately 2286 km^2^ of the total catchment area and has a total length of about 122.7 km. The Johor River’s headwater originates from the slopes of east Kluang and Gunung Belumut, which then moves south and discharges into the Straits of Johor. The Johor River has two major tributaries, which are the Sayong River and the Linggiu River. The streamflow station at Rantau Panjang (1,737,551, CA = 1130), as can be seen in Fig. 1, is among the main hydrometric stations and is situated downriver of a significant township. There are two gauges along the Rantau Panjang stream. One gives sediment measurement (No. 1737551), and the other measures river flow discharge (No. 1737451). Measurements of these two parameters are obtained on a daily basis from 1988 to 1998. The sediment and river flow discharge measurements have been utilized in this study. The data is illustrated in the sediment vs. time and discharge vs. time scatter plots in Figs. 2 and 3, respectively, while descriptive analyses of the sediment and discharge data can be seen in Table 1. Because of river water quality is a concern, the proposed research is essential. Urbanization and land-use practices have complicated the situation for the investigated study area^36,37^. Therefore, this investigating a reliable tool to predict the sedimentation with high precision for better surface water management.

**Table 1:** Descriptive data analysis of sediment and discharge for the Johor River basin from 1988 to 1998.

Parameter	Sediment	Discharge
**Descriptive data analysis**		
Mean	185.40	35.30
Standard error	4.10	0.58
Median	108.00	26.72
Mode	23.00	16.19
Standard deviation	259.23	36.76
Sample variance	67,200.04	1351.46
Kurtosis	31.22	73.49
Skewness	4.52	6.48
Range	3347.40	648.82
Minimum	5.60	1.84
Maximum	3353.00	650.66
Sum	740,690.70	141,007.70
Count	3995.00	3995.00

### Input sensitivity

One of the main tasks in machine learning is to choose input variables that have an impact on the output. A good understanding of the underlying process and statistical analysis of inputs and outputs are required to find a suitable model that links the inputs with the outputs. Usually, sediment is affected by the discharge and history its values and history of observed sediment values. There are three scenarios:

1. A history of the discharge as input for forecasting future sediment in scenario (1) in Eq. (1):
  1 $$SED_{t + n} = DIS_{t}$$
2. A history of sediment as input for forecasting future sediment in scenario (2) in Eq. (2):
  2 $$SED_{t + n} = SED_{t}$$
3. a history of sediment and discharge as inputs for forecasting future sediment in scenario (3) in Eq. (3):
  3 $$SED_{t + n} = DIS_{t} + SED_{t}$$

where SED_t_ is the sediment at time t, DIS_t_ is discharge at time t, n is a one (day, week, 10-days, month) ahead value until the seven (day, week, 10-days, month) ahead.

### Data partitioning

This section describes the experimental protocol and data partitioning process. Our dataset contains three sets of training, validation, and testing. The training set was employed to train the models, learn the patterns from the input, and tune their weights. At the same time, the validation set was used in the training stage to overcome the overfitting problem. On the other hand, a testing set was utilized to evaluate the models and calculate the performance metrics. The dataset was divided into two sets: training and testing with the rule 80/20. In this splitting, 80% of data, including the first years of our dataset, was assigned to the training set, while 20% of data, including the last years, was assigned to the testing set. After that, the training set was divided again with the same rule of 80/20 to get the final training set and validation set. This splitting between training and validation was done five times randomly using the 5-cross validation technique to select the best model between five models that produces the best evaluation metrics with the testing data.

### Models used for forecasting

In this paper, four models were used for forecasting, which include ElasticNet Linear Regression (ElasticNet LR)^38–40^, Multilayer Perceptron Neural Network (MLP NN)^41^, Extreme Gradient Boosting (XGB)^42^, and Long Short-Term Memory (LSTM)^43^.

Many methods were used to predict SSL in the literature review. The methods were divided into conventional ML and deep learning methods. In this study, we selected various baseline of ML methods to compare with our proposed LSTM. The selection was made considering various model structures and learning mechanisms in the models to get a fair comparison.

The training and testing for the LSTM model were carried out by using the TensorFlow framework on an NVIDIA GeForce GTX 1080 Ti GPU.

#### ElasticNet linear regression

ElasticNet LR is a regularization linear regression technique that is usually used to reduce overfitting in linear model^39^. Linear Regression is a well-known regression method, but this version was improved by adding regularization terms to loss function to improve model's predictions^45,46^. ElasticNet LR penalizes the least-squares method using an elastic net penalty. It combines two popular penalty functions, namely L1 and L2, to the loss function during training^39^. It was found to overcome the limitations of the lasso technique. ElasticNet is a hybrid of Lasso and Ridge Regression techniques and has the advantage of trading-off between Lasso and Ridge.

4 $$\hat{\beta } = argmin_{\beta } \left( {y - X \beta^{2} + \lambda_{2 } \beta^{2} + \lambda_{1 } \beta_{1} } \right)$$

$\hat{\beta }$ are optimal weights to minimize the loss function which is represented by the squared difference between the actual and forecasted output with two regularization terms added. These terms are L2 penalty $\lambda_{2 } \beta^{2}$ and L1 penalty $\lambda_{1 } \beta_{1}$ with two parameters $\lambda_{1 }$ and $\lambda_{2 }$ to be tuned. The values of parameters $\lambda_{1 }$ and $\lambda_{2 }$ should be selected carefully to improve the prediction performance. Various values of parameters $\lambda_{1 }$ and $\lambda_{2 }$ were evaluated to find optimal values as shown in the “Results and discussion” section.

#### MLP neural network

MLP neural network is a network with several layers, and nonlinear activation functions^38,47^. The parameters of this network are tuned iteratively (800 iterations) to find optimal ones. Several hyperparameters were tested to find the best ones. These hyperparameters are:

1. activation: logistic, tanh, or relu.
2. solver : lbfgs, adam, or sgd.
3. learning_rate^48^: constant, invscaling, or adaptive.

The neural network architecture is defined by the number of hidden layers, the number of nodes in each hidden layer, and the type of activation function^49,50^. In this study, different MLP NN architectures were evaluated by changing the number of hidden layers and the number of nodes in layers. The final best architecture that gave the best metrics in terms of R^2^, MAE, RMSE was as follows:

- the input layer with the number of nodes equals historical values of sediment and discharge
- the output layer, which has one node for sediment forecasting
- one hidden layer with 100 nodes.

#### Extreme gradient boosting (XGB)

XGB is an end-to-end tree learning system. It runs more than ten times faster than existing solutions on a single machine and scales to a large number of examples in memory-limited resources^38,51^. Various algorithmic optimizations are behind the scalability of XGB. It uses a gradient descent algorithm to minimize the loss and a regularization technique to control the over-fitting^42^.

#### Long short-term memory

**Figure 4:**
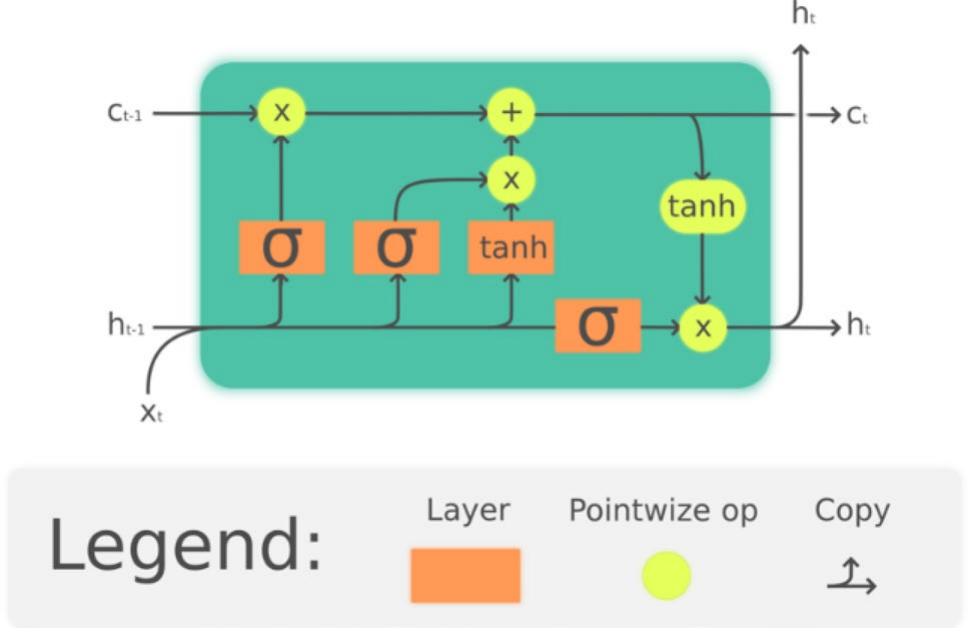
LSTM cell^54^.

LSTM is a special type of Recurrent Neural Network (RNN) that is used for long-range sequence modeling^39,52^. LSTM has a memory cell, as shown in Fig. 4, which acts as an accumulator of state information, supported by control gates. The advantage of this structure is that it speeds down the gradient vanishing. LSTM network was found to capture temporal correlations^53^.

In this study, a series of discharge and sediment observations were applied to LSTM. The parameters of LSTM were tuned iteratively to fit the data.

To validate the optimal structure of LSTM, various architectures, including a number of LSTM layers, number of nodes in each layer, number of fully connected layers, types of activation function, and number of dropout layers, were tested and evaluated to select the best architecture that gives the best evaluation metrics. The best architecture of the proposed LSTM model consists of the following layers:

1. LSTM with 64 nodes and ReLU activation function
2. Dropout with 0.1
3. Fully connected layers with 32 nodes and ReLU activation function
4. Dropout with 0.1
5. Fully connected layers with 1 node and Linear activation function

Various hyperparameters, including learning rate, loss function, optimizer, percentage of dropout, batch size, and a number of epochs, were tested and evaluated to select the optimal hyperparameters that give the best evaluation metrics. The final hyperparameters were as follows:

1. The learning rate used to train the LSTM model was set to 0.002 to balance the speed of learning (done in 400 epochs) and avoidance of undesirable divergence.
2. The batch size was set to 8 to balance the speed of convergence and good performance.
3. The number of epochs was set to 400.
4. MAE and MSE loss functions were evaluated. It was found that the MAE loss function can be minimized better using the Adam optimizer.

In summary, models described earlier were utilized for forecasting the sediment using traditional machine learning methods as baseline models and LSTM as the proposed model. The behavior of sediment is affected by different factors such as the history of discharge and sediment.

### Performance evaluation

In this section, five standard evaluation metrics such as coefficient of determination (R2), mean absolute error (MSE), root mean square error (RMSE), relative absolute error (RAE), and relative squared error (RSE) were utilized. The larger value of R^2^ refers to the better prediction performance of the model. However, R^2^ is not enough to determine whether the coefficient prediction is biased or not. Therefore, to further investigate if a regression model provides a good fit to our data, other error metrics were used, such as RAE, RSE, MAE, and RMSE, to find the error or difference between the actual and predicted outcome. The smaller value of RSE, RAE, MAE., and RMSE refers to the model's better prediction performance. The drawback of RMSE is that it is more sensitive to big errors and outliers than MAE. On the other hand, RSE was found to solve the RMSE drawback of sensitivity to the mean and scale of predictions. In addition, we used absolute error distribution (A.E.) plots to evaluate the prediction models by calculating the frequency of absolute errors in four scenarios.

Using this bag of previous metrics can help us to make completed evaluations of the proposed and baseline models addressing all previously mentioned drawbacks of individual metrics.

This section describes the performance indicators as follows:

1. Coefficient of determination (R^2^) represents a statistical measure to study the correlation (trend) between the actual and the forecasted output. R^2^ = 0 means the model is random. R^2^ = 1 means that the model fits data perfectly.
  5 $$R^{2} = \frac{{\mathop \sum \nolimits_{i = 1}^{n} \left( {y - \overline{y}} \right) \left( {\hat{y} - \overline{{\hat{y}}} } \right)}}{{\sqrt {\mathop \sum \nolimits_{i = 1}^{n} \left( {y - \overline{y}} \right)^{2 } } \mathop \sum \nolimits_{i = 1}^{n} \left( {\hat{y} - \overline{{\hat{y}}} } \right)^{2} }}$$
2. Mean absolute error (MAE): it represents the absolute error between the actual and the forecasted output.
  6 $$MAE = \frac{1}{n}\mathop \sum \limits_{i = 1}^{n} \left| {y - \hat{y}} \right|$$
3. Root Mean Square Error (RMSE): it represents the root of average squared error between the actual and the forecasted output.
  7 $$RMSE = \sqrt {\frac{{\mathop \sum \nolimits_{i = 1}^{n} \left( {y - \hat{y}} \right)^{2} }}{n}}$$
4. Relative absolute error (RAE): it stands for a normalized sum of absolute differences between the actual and the forecasted outputs.
  8 $$RAE = \frac{{\mathop \sum \nolimits_{i = 1}^{n} \left| {y - \hat{y}} \right|}}{{\mathop \sum \nolimits_{i = 1}^{n} \left| {y - \overline{y}} \right|}}$$
5. Relative squared error (RSE): it stands for a normalized sum of squared differences between the actual and the forecasted outputs.
  9 $$RSE = \frac{{\mathop \sum \nolimits_{i = 1}^{n} \left( {y - \hat{y}} \right)^{2} }}{{\mathop \sum \nolimits_{i = 1}^{n} \left( {y - \overline{y}} \right)^{2} }}$$

where n is the number of samples, y is actual output, $\hat{y}$ is a is forecasted output, $\overline{y}$ is an average of actual output.

### Autocorrelation function (ACF)

**Figure 5:**
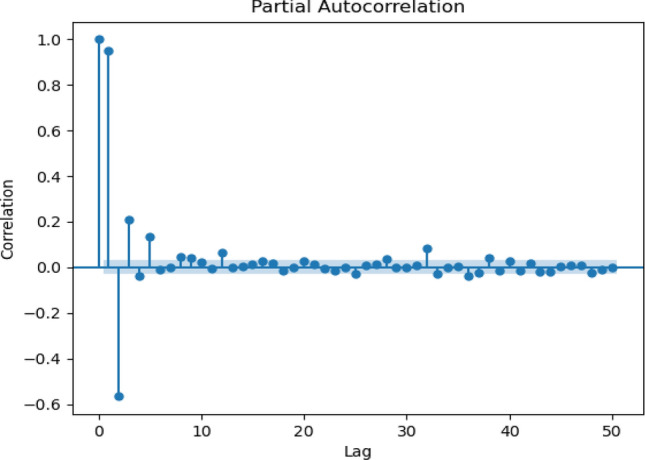
Partial autocorrelation for daily scenario.

**Figure 6:**
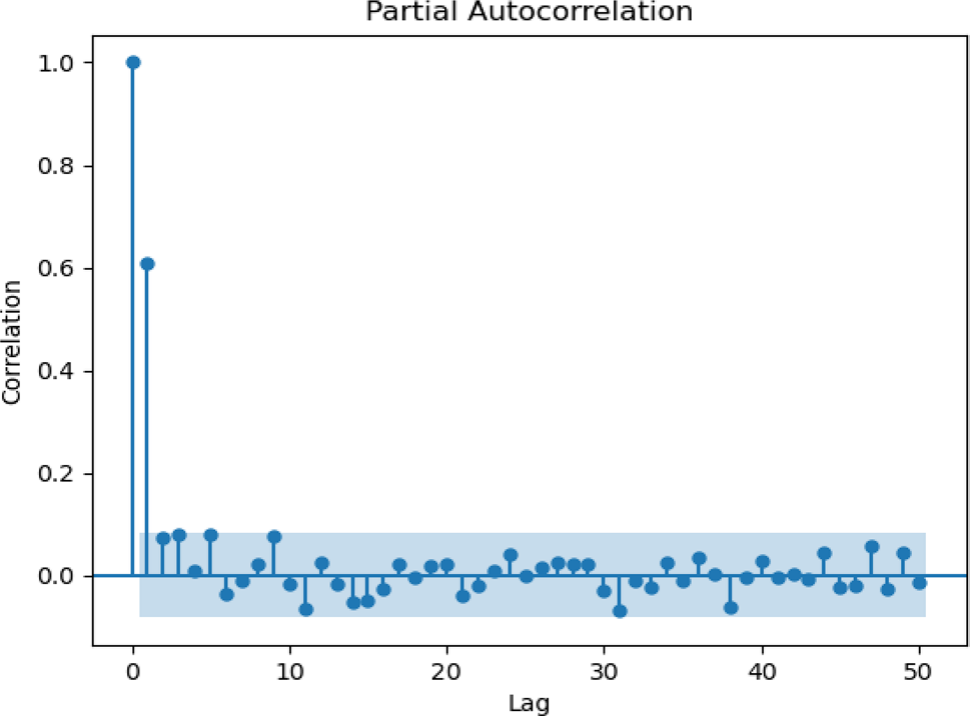
Partial autocorrelation for the weekly scenario.

**Figure 7:**
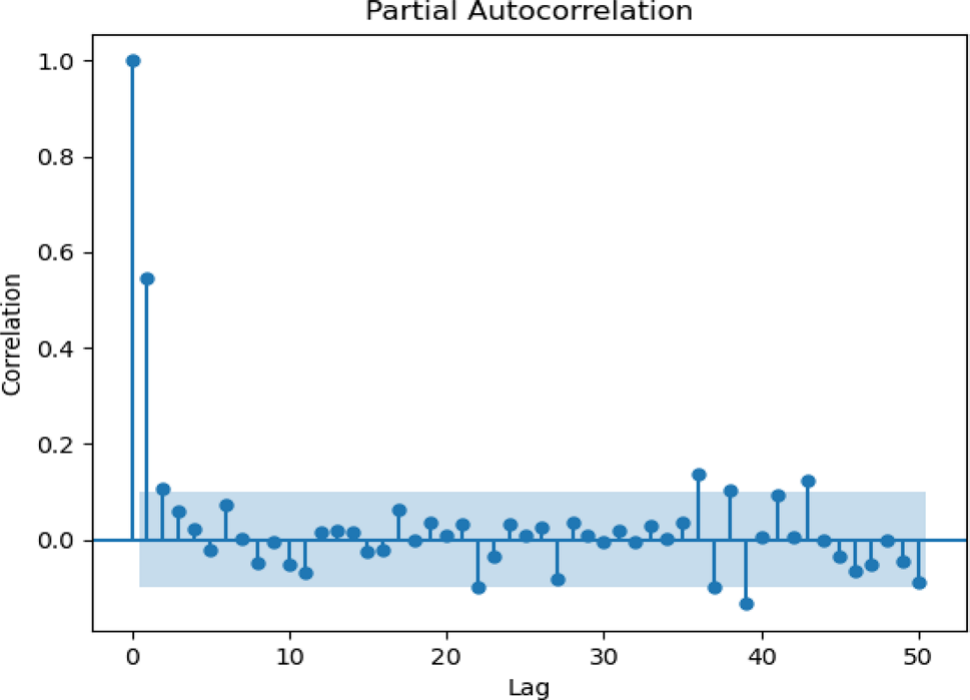
Partial autocorrelation for 10_days scenario.

**Figure 8:**
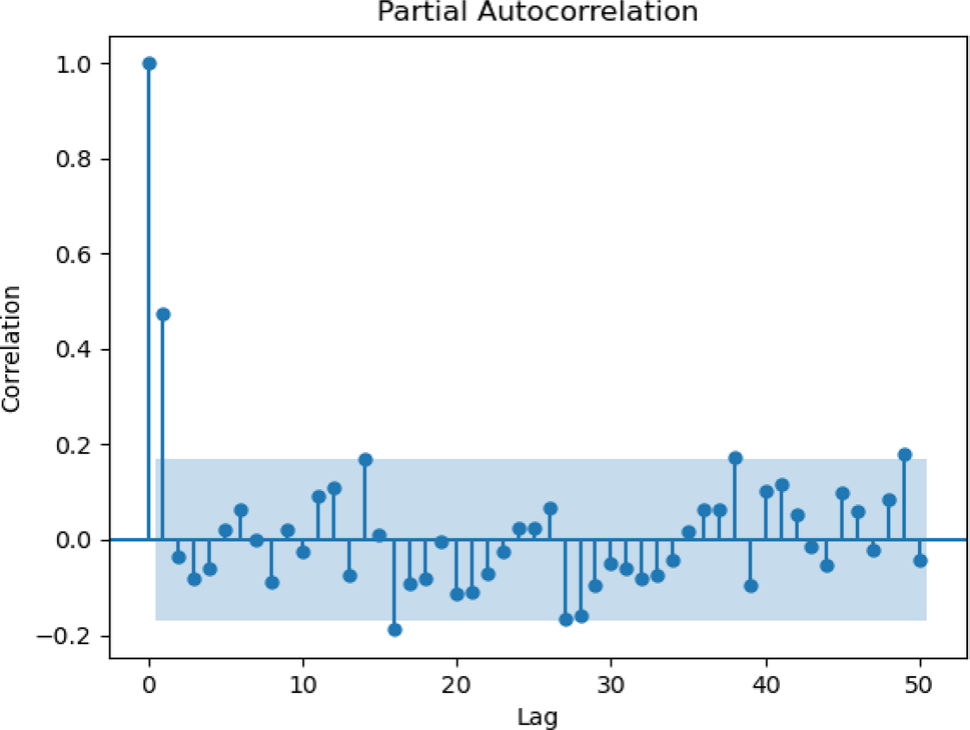
Partial autocorrelation for the monthly scenario.

ACF is an effective analytical tool used with time series forecasting and analysis^55^. This function aims to measure the statistical relationships between observations in a single data series. In other words, ACF defines the relation between the current and past values of the observation. Additionally, it finds the correlations taking into account components like trends, seasonality, cyclic, and residual. Figures 5, 6, 7 and 8 show four scenarios of historical patterns in ACF, including daily, weekly, 10-days, and monthly.

The correlogram shows that the data have seasonal dependencies and the same pattern over the years. For daily, the analysis took historical data from the year 1988 until the year 1998. Figure 5 shows data observed for 50 days starting from January 1988. Figure 6 illustrates 50 lags of weeks, where each lag represents one week. Meanwhile, a 10-days correlogram is shown in Fig. 7 for 50 lags of 10 days. In other words, each lag represents 10 days. When the lag gives a high value of sediment above the upper line, it means that the output at this lag has a high correlation. Figure 8 shows 50 lags of monthly sediment for 50 months.

**Figure 9:**
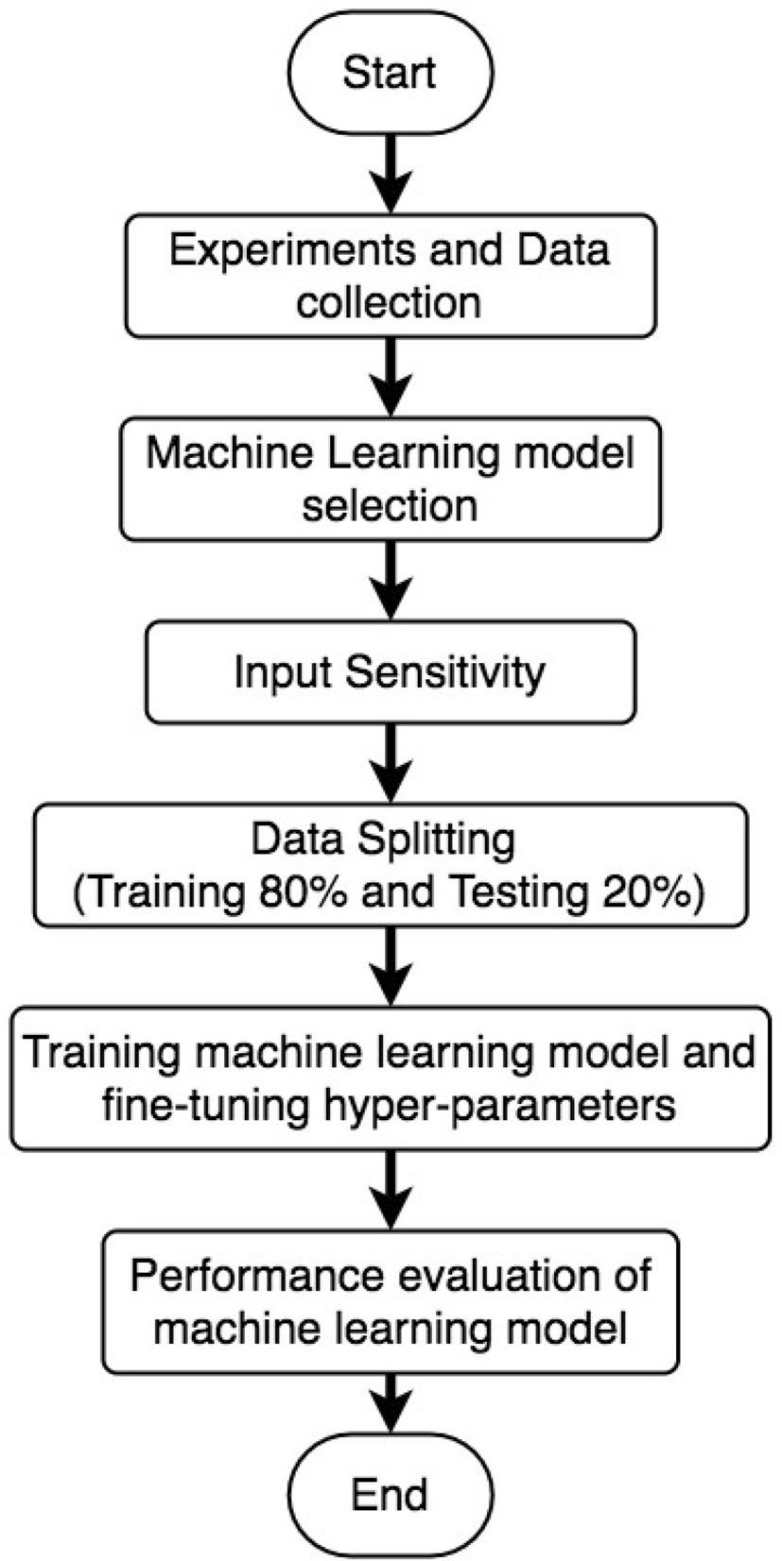
Flow chart of the proposed methodology to forecast sediment using machine learning models.

To summarize the training and performance evaluation process a flow chart has been developed, which is illustrated in Fig. 9. The flow chart shows the step-by-step process followed in the methodology.

## Results and discussion

**Figure 10:**
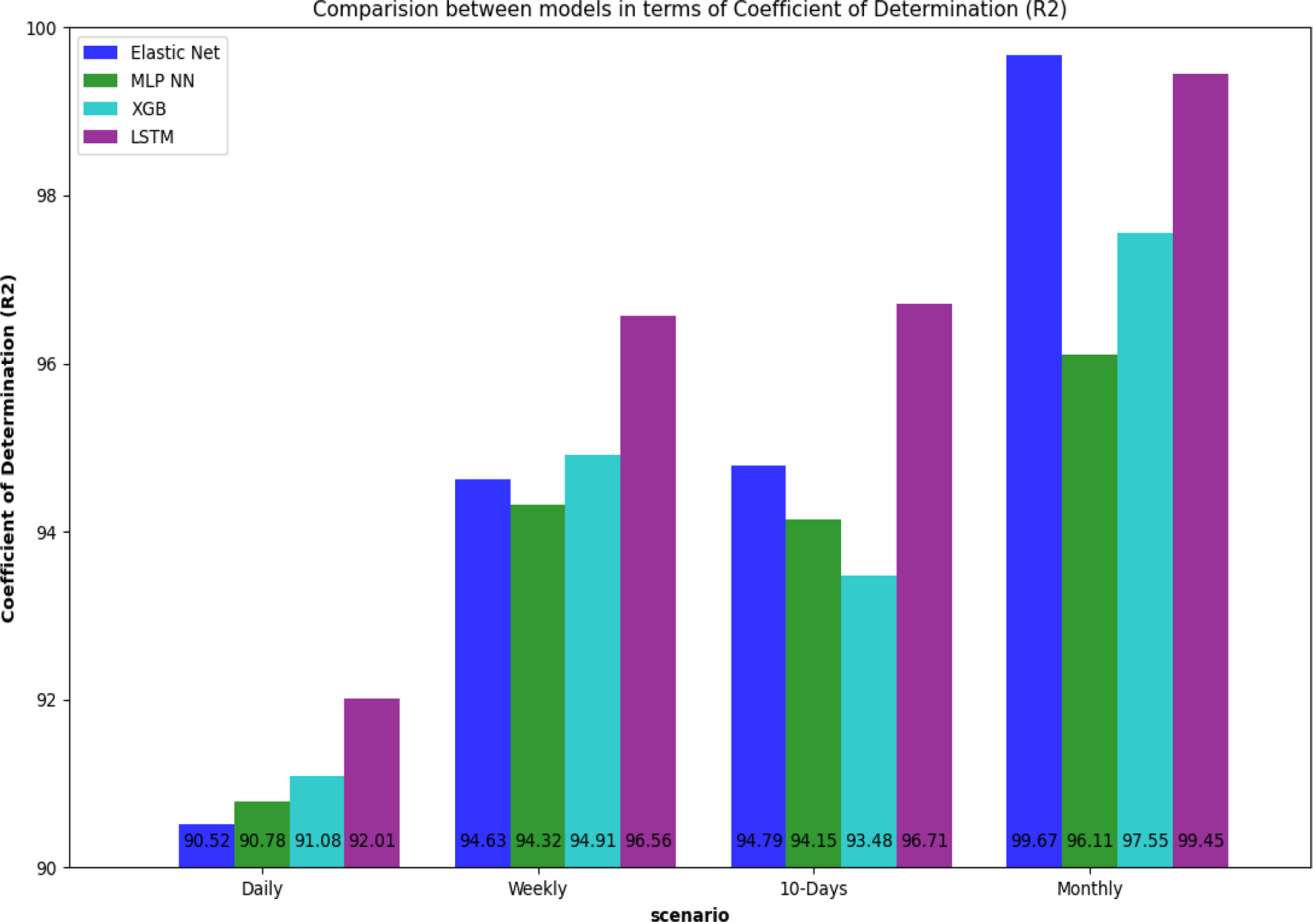
Coefficient of determination for four scenarios: daily, weekly, 10-days, monthly for four models: Elastic Net, MLP NN, XGB, and LSTM.

**Figure 11:**
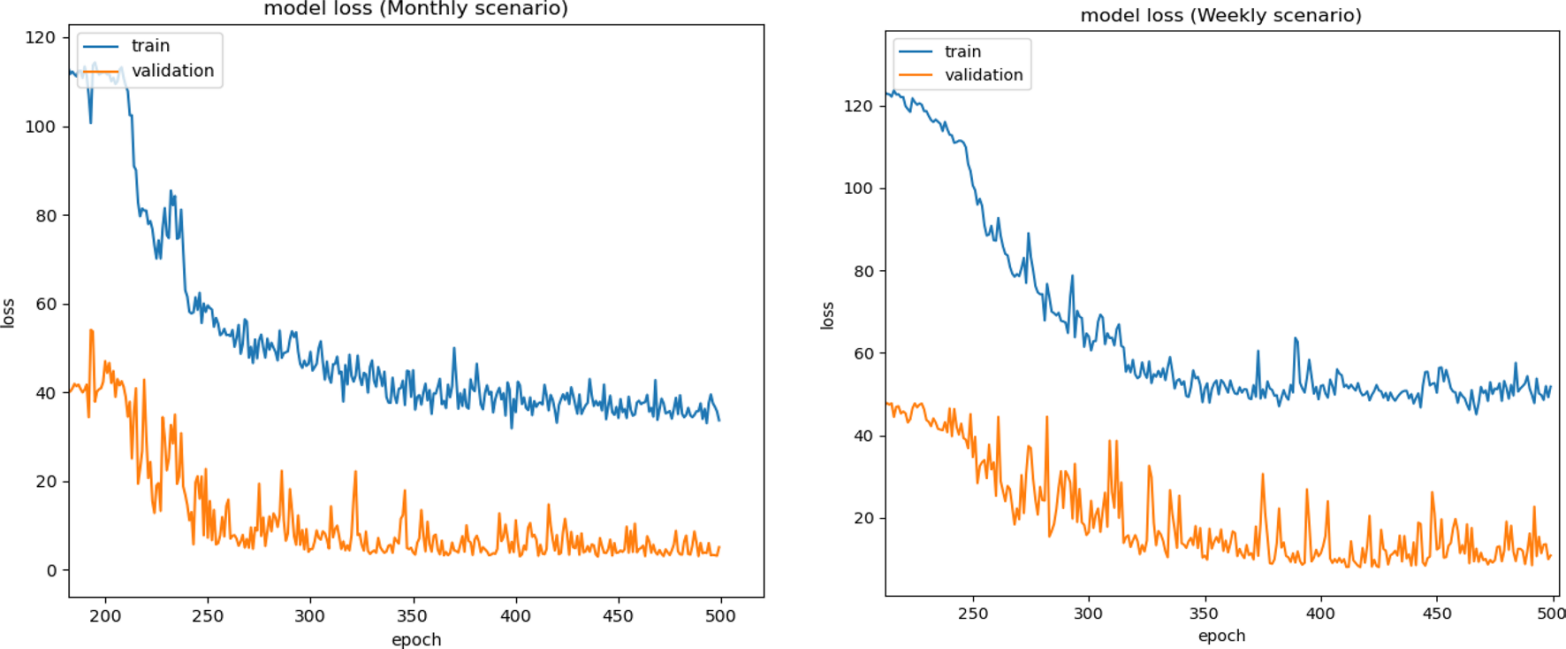
Learning curves for LSTM in weekly (on the right) and monthly (on the left) scenarios.

The first experiment was aimed to forecast the sediment for one day ahead using four machine learning models, including ElasticNet Linear Regression (ElasticNet LR)^38^, Multilayer Perceptron Neural Network (MLP NN), Extreme Gradient Boosting (XGB)^47^, and Long Short-Term Memory (LSTM). To evaluate the performance of the proposed models, Coefficient of Determination (R^2^), Mean Absolute Error (MAE), Root Mean Square Error (RMSE), Relative Absolute Error (RAE), and Relative Squared Error (RSE),) were used. The hyperparameters of the models were tuned to optimize the models to give the best results. Table 2 and Fig. 10 summarize each model's performance metrics for four scenarios, including daily, weekly, 10-days, and monthly scenarios. In these four scenarios. The data were divided into daily (values of discharge and sediment for each day), weekly (average values of discharge and sediment for each week), 10-days (average values of discharge and sediment for each 10-days), and monthly (average values of discharge and sediment for each month). The objective of demonstrating these four scenarios is to study the data variation and explore the hidden patterns that the model should be able to learn. It was found that monthly data in the monthly scenario have patterns that can be learned well and generalized to future examples to predict future SSL. In this experiment, LSTM outperformed other baseline solutions such as ElasticNet LR, MLP NN, and XGB in all scenarios. In the monthly scenario, even the dataset used for training has a small size, LSTM, a data-hungry deep learning model, could compete ElasticNet LR. LSTM obtained R^2^ of 92.01%, 96.56%, 96.71%, and 99.45% in daily, weekly, 10-days, and monthly scenarios, respectively. The learning curves of LSTM for monthly and weekly scenarios were shown in Fig. 11.

**Table 2:** Performance indicators for four scenarios: daily, weekly, 10-days, monthly, and four models: Elastic Net, MLP, XGB, and LSTM.

Model	Elastic net baseline	MLP NN baseline	XGB baseline	LSTM proposed
**Scenario**				
**Daily**				
MAE	15.52	14.43	14.15	12.55
RMSE	25.053	24.49	24.19	22.92
RAE	0.264	0.249	0.243	0.216
RSE	0.094	0.092	0.089	0.079
**Weekly**				
MAE	11.26	11.56	11.02	8.601
RMSE	15.4	15.08	14.92	11.84
RAE	0.236	0.254	0.233	0.187
RSE	0.054	0.057	0.051	0.034
**10_days**				
MAE	10.64	11.45	11.22	8.088
RMSE	13.88	14.7	15.26	11.04
RAE	0.24	0.268	0.267	0.183
RSE	0.052	0.058	0.065	0.033
**Monthly**				
MAE	2.713	6.642	6.269	2.447
RMSE	3.089	8.644	7.685	3.236
RAE	0.062	0.203	0.167	0.075
RSE	0.003	0.039	0.024	0.005

The objective of utilizing four scenarios is to explore various patterns from the data. In the daily scenario, our used data are suffering from big variations and noise, which makes the process of pattern learning more complex compared to other scenarios. In weekly and 10-days scenarios, the variation in data was reduced, making the learning of pattern more efficient, and the performance metrics were improved compared to the daily scenario. Lastly, in the monthly scenario, the learning model's performance got remarkable improvement in terms of R2, RSE, RAE, RMSE, and MAE, as shown in Table 2 and Fig. 10.

**Figure 12:**
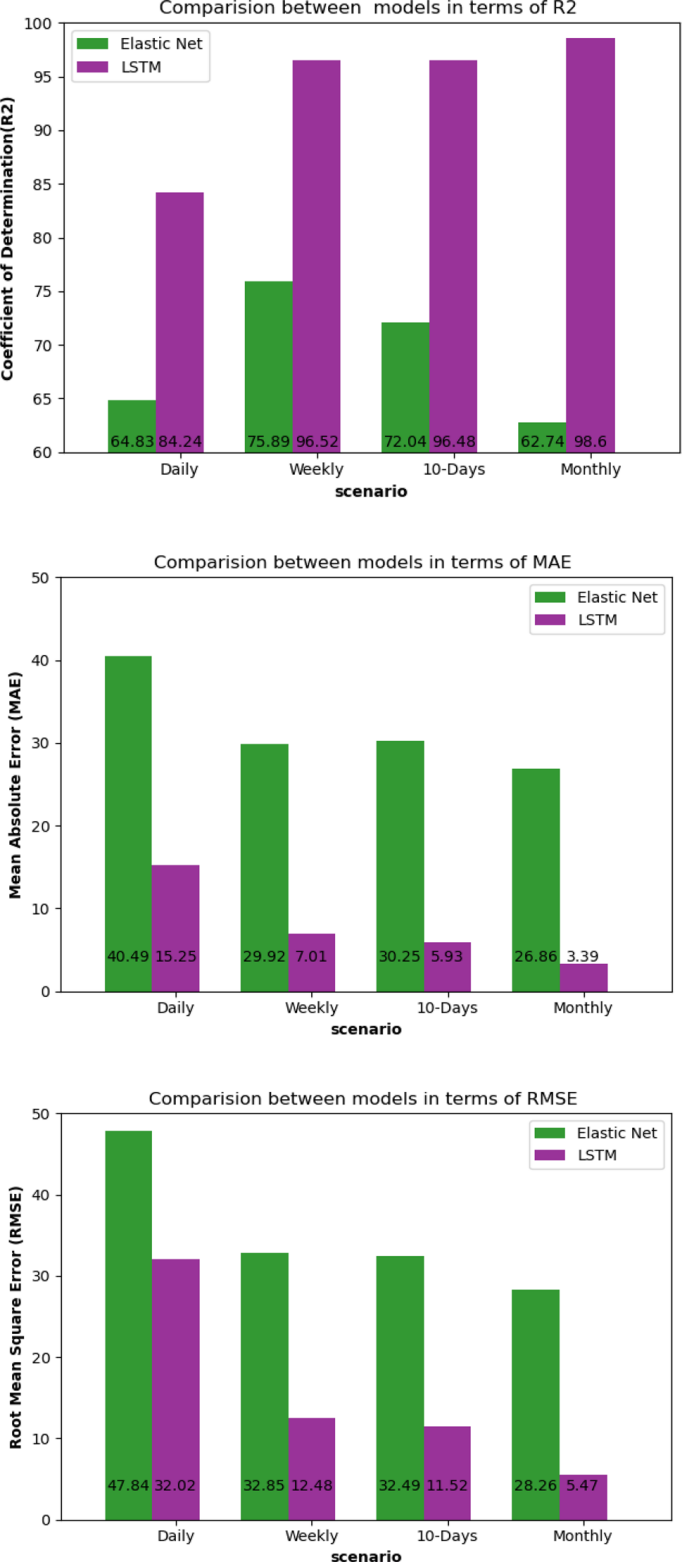
Comparison between Elastic Net L.R. and LSTM in terms of R^2^, MAE, RMSE for four scenarios when input is discharge at time t.

The second experiment was aimed to study all possible combinations of inputs to investigate and analyze the input sensitivity on SSL prediction, which is a significant stage in building a prediction model. The evaluation was done using evaluation metrics. Various inputs of discharge and sediment were selected to find the optimal combination that gives the best Coefficient of Determination in each model and each scenario. Table 3 summarizes the R^2^ for each model for four scenarios, including daily, weekly, 10-days, and monthly scenarios. The best obtained R^2^ values were shown in bold font in Table 3. It was found that combining the history of inputs of both discharge and sediment outperformed taking the only history of discharge or sediment to predict the future sediment in almost all scenarios. Additionally, ignoring the input discharge and using only previous sediment values to predict future sediment is only acceptable in daily scenarios and produces bad forecasting results in other scenarios. Moreover, it was found that using only discharge as input at time t to predict sediment at time t is possible by using the LSTM model, which obtained R^2^ of 84.24%. 96.52%, 96.48%, and 98.6% in daily, weekly, 10-days, and monthly respectively. On the other hand, ElasticNet was not able to provide good R^2^, MAE, and RMSE. Figure 12 illustrates the R^2^, MAE, and RMSE for Elastic Net L.R. and LSTM.

**Table 3:** Sensitivity analysis of inputs for four scenarios: daily, weekly, 10-days, and monthly and four models: Elastic Net, MLP, XGB, and LSTM.

Model	Inputs		Coefficient of determination R^2^			
			Scenario			
	Sediment	Dischrge	Daily	Weekly	10-Day	Monthly
Elastic Net baseline	Yes	No	0.8398	− 0.0953 < 0	− 0.3032 < 0	− 0.9204 < 0
MLP NN baseline	Yes	No	0.8218	− 0.1414 < 0	− 0.1588 < 0	− 0.0878 < 0
XGB baseline	Yes	No	0.8407	0.0151	− 0.1221 < 0	− 0.4091 < 0
LSTM proposed	Yes	No	0.8121	0.1307	0.0778	0.2406
Elastic Net baseline	No	Yes	0.7494	0.8437	0.9056	0.9092
MLP NN baseline	No	Yes	0.8775	0.8931	0.9376	**0.9611**
XGB baseline	No	Yes	0.7933	**0.9491**	**0.9348**	0.9622
LSTM proposed	No	Yes	0.8972	0.9598	0.9603	**0.9945**
Elastic Net baseline	Yes	Yes	**0.9052**	**0.9463**	**0.9479**	**0.9967**
MLP NN baseline	Yes	Yes	**0.9078**	**0.9432**	**0.9415**	0.9443
XGB baseline	Yes	Yes	**0.9108**	0.9333	0.9191	**0.9755**
LSTM proposed	Yes	Yes	**0.9201**	**0.9656**	**0.9671**	0.9833

**Figure 13:**
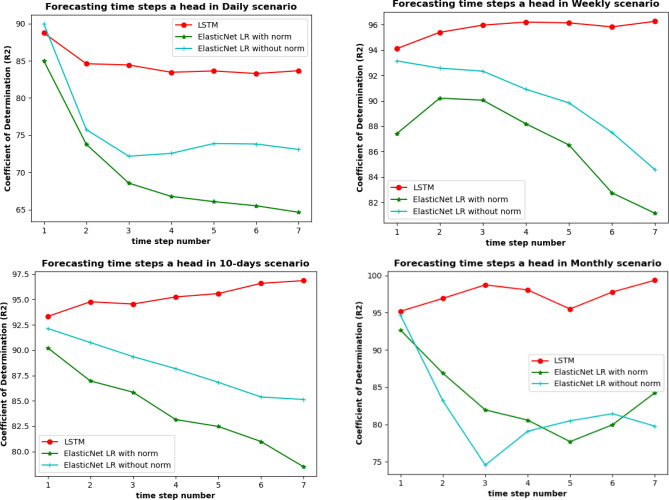
Coefficient of determination for the four scenarios four two models to predict from one to seven days ahead.

The third experiment aimed to demonstrate the model's ability to do forecasting multiple time steps ahead, such as few days ahead and few months ahead. This experiment is essential to study the proposed model's generalization capability to learn new patterns from new future cases. The comparison between LSTM and ElasticNet LR (with and without normalization) for four scenarios in terms of R^2^ for one to seven-time steps ahead was made. The time step includes day, week, 10-days, or month. Table 4 shows the R^2^ for LSTM and ElasticNet LR. The best obtained R^2^ values were shown in bold font. It can be seen clearly in scenario one that LSTM has outstanding performance in predicting the sediment for one day and seven days ahead. In addition, LSTM outperformed ElasticNet LR and produced better forecasting results in all scenarios. Figure 13 illustrates the R^2^ for Elastic Net L.R. and LSTM in various time steps ahead. SED + 1 for all scenarios shows that the model fits the data better than the next week of sediment (SED + 2) until SED + 7. The results indicate that LSTM can be used to predict the expected changes in sediment one week ahead.

**Table 4:** Coefficient of determination for the four scenarios four two models to predict from one to seven days ahead.

No	Outputs	Coefficient of determination R^2^%		
		ElasticNet without normalization	ElasticNet with normalization	LSTM proposed
Daily	SED + 1	**89.95**	84.99	88.74
Daily	SED + 2	75.76	73.78	**84.61**
Daily	SED + 3	72.17	68.56	**84.44**
Daily	SED + 4	72.56	66.77	**83.46**
Daily	SED + 4	73.88	66.07	**83.64**
Daily	SED + 6	73.82	65.51	**83.29**
Daily	SED + 7	73.08	64.64	**83.67**
Weekly	SED + 1	93.15	87.4	**94.11**
Weekly	SED + 2	92.58	90.21	**95.4**
Weekly	SED + 3	92.34	90.05	**95.97**
Weekly	SED + 4	90.91	88.19	**96.21**
Weekly	SED + 4	89.84	86.52	**96.15**
Weekly	SED + 6	87.5	82.74	**95.83**
Weekly	SED + 7	84.56	81.13	**96.27**
10-days	SED + 1	92.14	90.23	**93.32**
10-days	SED + 2	90.75	86.96	**94.77**
10-days	SED + 3	89.36	85.85	**94.55**
10-days	SED + 4	88.17	83.15	**95.25**
10-days	SED + 4	86.82	82.47	**95.58**
10-days	SED + 6	85.38	80.98	**96.59**
10-days	SED + 7	85.13	78.48	**96.86**
Monthly	SED + 1	94.69	92.68	**95.19**
Monthly	SED + 2	83.18	86.88	**96.92**
Monthly	SED + 3	74.55	81.96	**98.73**
Monthly	SED + 4	79.09	80.59	**98.06**
Monthly	SED + 4	80.49	77.7	**95.5**
Monthly	SED + 6	81.45	79.96	**97.78**
Monthly	SED + 7	79.77	84.25	**99.35**

**Figure 14:**
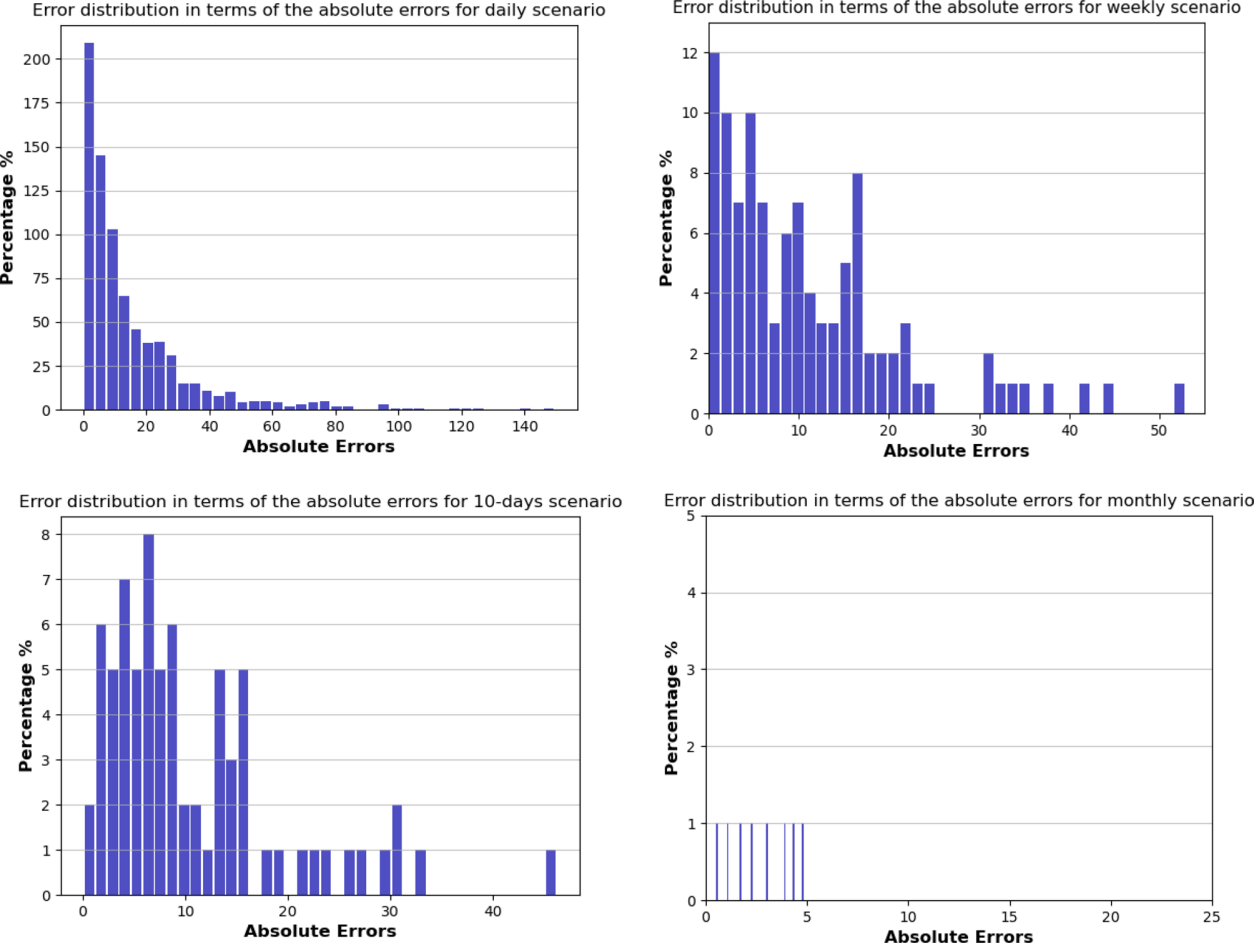
Error distribution in terms of the absolute errors (A.E.) for four scenarios: daily, weekly, 10-days, and monthly.

Figure 14 visualizes absolute error (A.E.) distribution to evaluate the proposed prediction model by calculating the frequency of absolute errors in four scenarios. It is evident that the points are gathered around low values of error with high frequencies. The absolute errors in the monthly scenario have values under 5.

**Figure 15:**
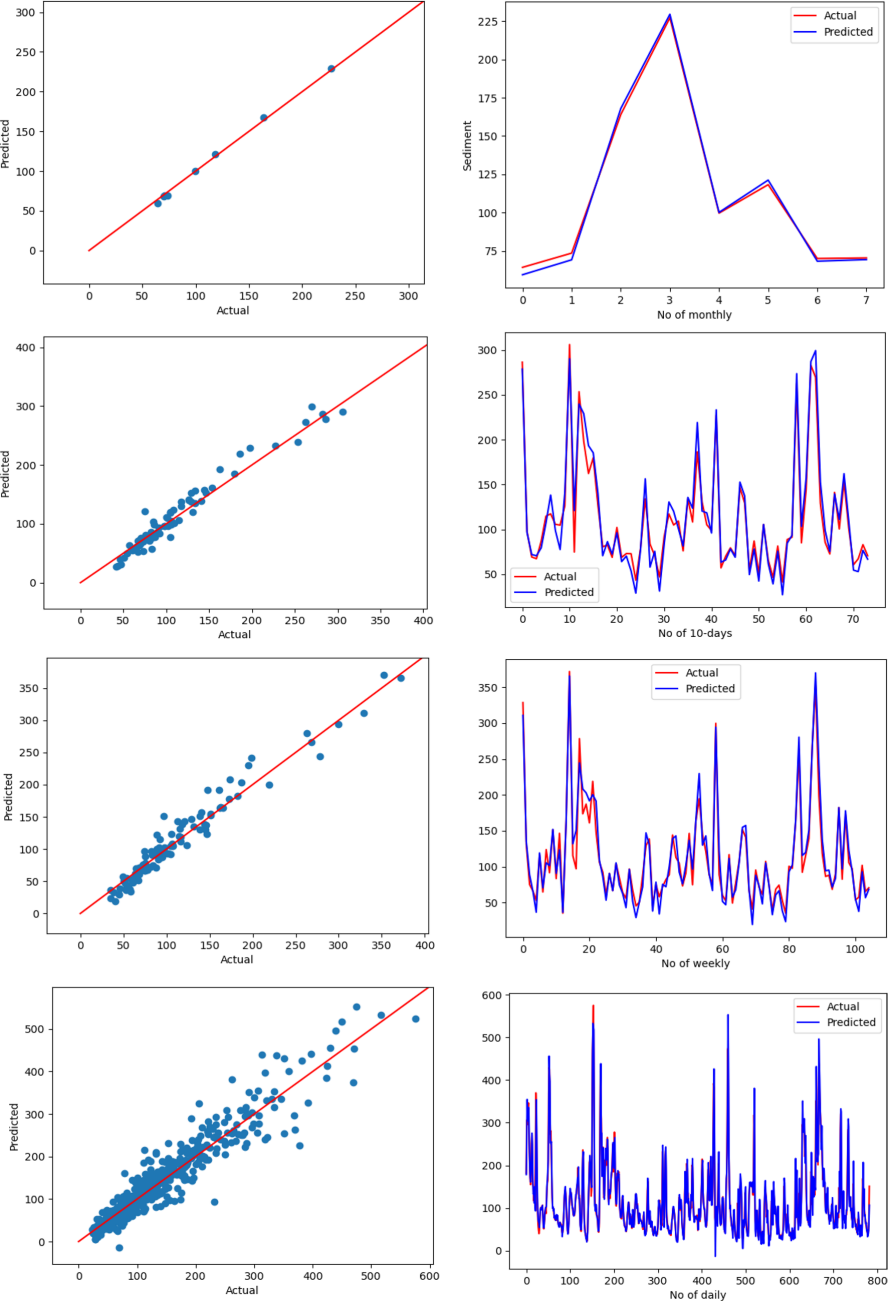
Scatter plot of actual sediment versus forecasted sediment for the proposed model for four scenarios: monthly (first row), 10 days (second row), weekly (third row), daily (fourth row).

**Figure 16:**
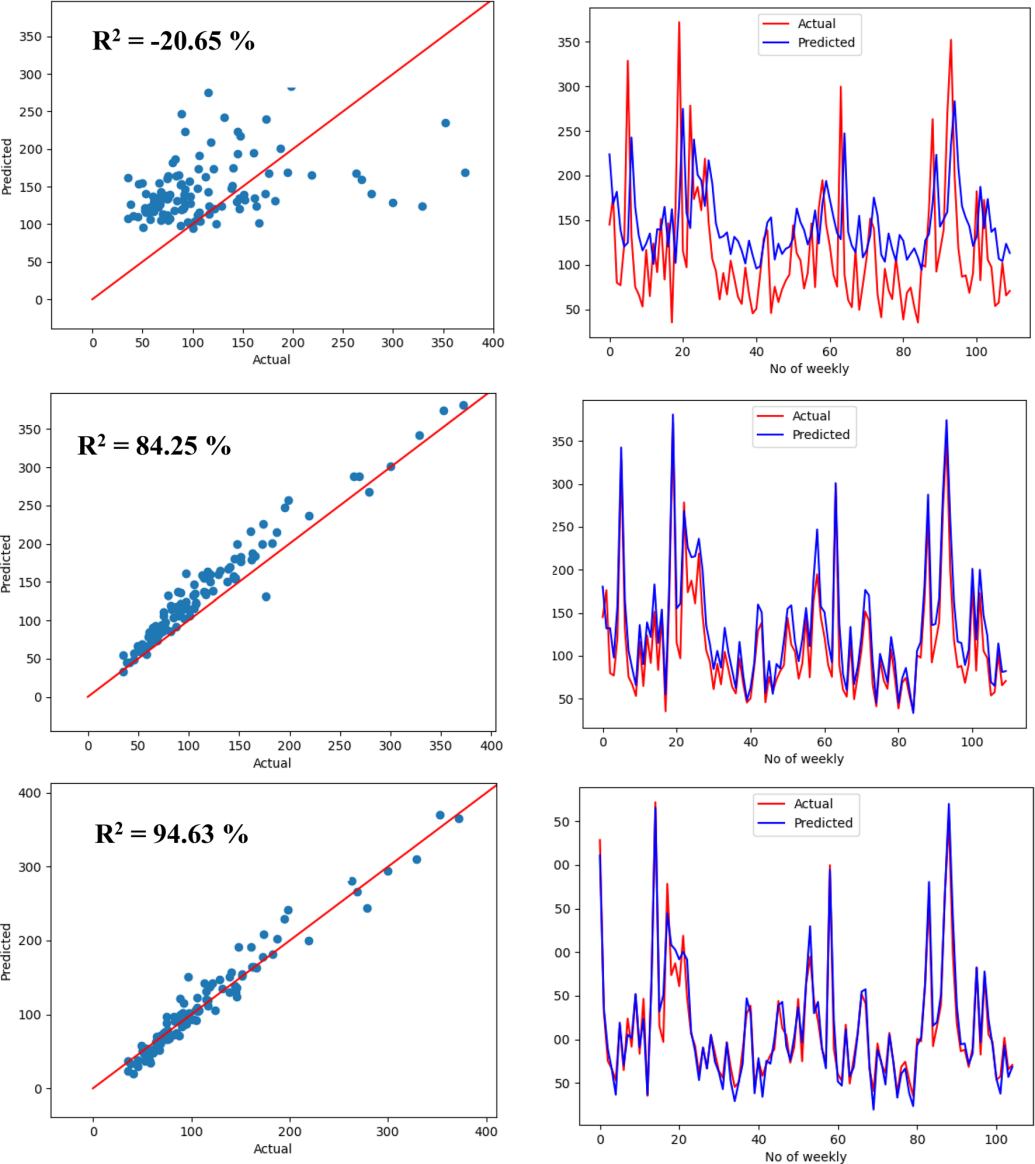
Scatter plot and signal plot of actual sediment versus forecasted sediment for ElasticNet LR for the weekly scenario for different inputs: sediment only (first row), discharge only (second row), sediment and discharge (third row).

**Figure 17:**
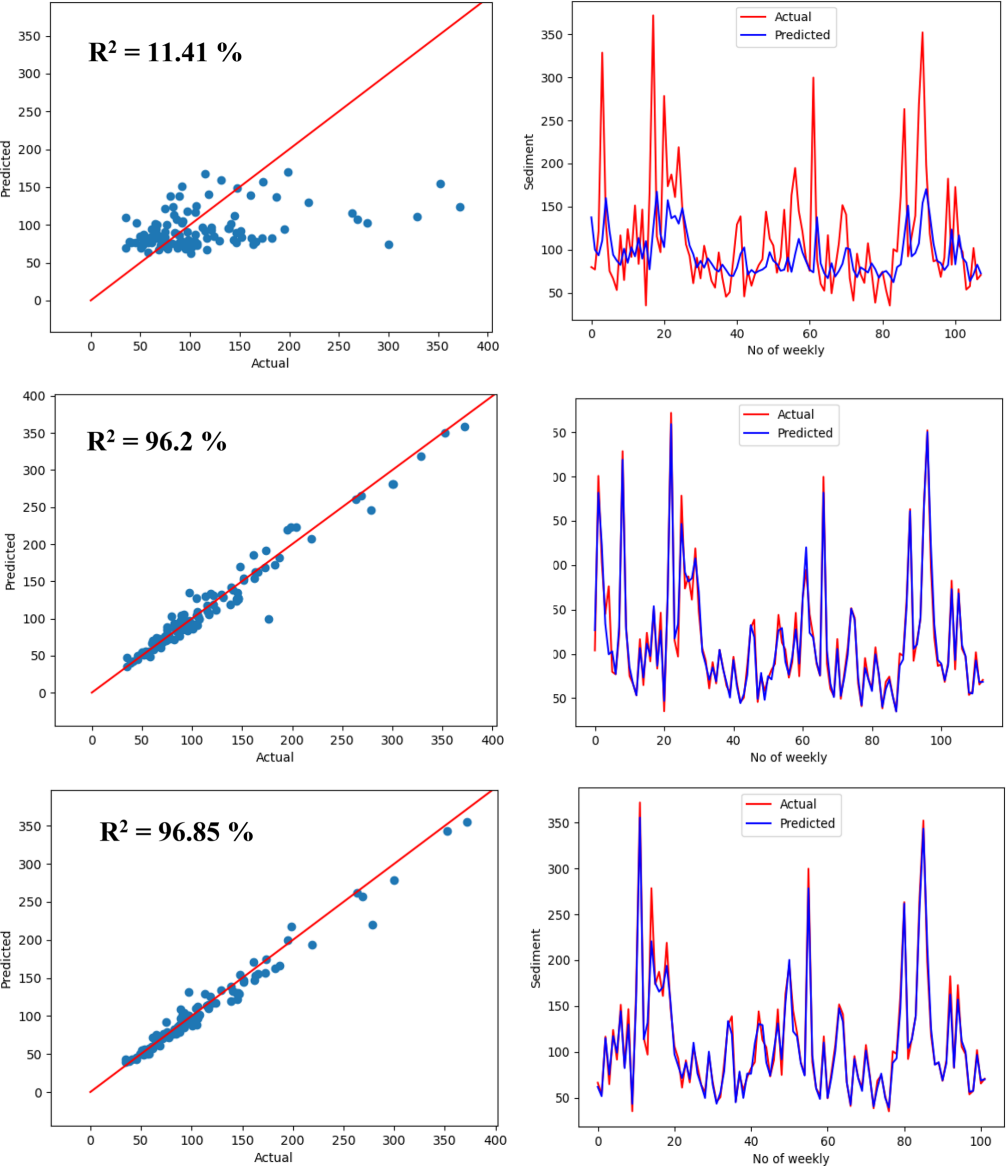
Scatter plot and signal plot of actual sediment versus forecasted sediment for LSTM for the weekly scenario for different inputs: sediment only (first row), discharge only (second row), sediment and discharge (third row).

To visually explore how R^2^ values represent the scatter around the regression line, Figs. 15, 16, and 17 plot the fitted values by observed values. The scatter plots of the proposed LSTM model were shown in Fig. 15. The figure illustrates the scatter plot of actual sediment versus forecasted sediment for four scenarios: daily, weekly, 10 days, and monthly. In addition, the signals of actual and forecasted sediment were shown in this figure. It was evident that there is a big match between the actual and forecasted sediment in all scenarios and specifically the monthly one.

Having two parameters result in three scenarios for input sensitivity study. These three scenarios aim to study the impact of inputs on the learning model. In this experiment, it was found that using both history of discharge and sediment can improve the evaluation metrics compared to using only one input. Additionally, discharge input with its histories plays a significant role in predicting SSL. On the contrary, utilizing only previous historical data of sediment is not enough and not efficient to predict future sediment. In Figs. 16 and 17, using both sediment and discharge as inputs leads to better matching between actual sediment and forecasted sediment than using sediment discharge only. For the weekly scenario, using sediment only gives the worst fit of the two models. On the other hand, using discharge only in both models can give a good fit but less than using both sediment and discharge.

Table 5 shows the impact of two factors: alpha and in ElasticNet LR. The coefficient of determination was calculated for various values of alpha and l_1__ratio. It was found that when alpha = 0.1 and l1_ratio = 1, R^2^ has the best value of 94.09%.

**Table 5:** Coefficient of determination for various values of alpha and l_1__ratio for ElasticNet linear regression for weekly scenario.

l1_ratio	Alpha	0.001	0.01	0.1	1	10	100	1000
0		90.85	51.32	− 1.21	− 11.8	− 12.96	− 13.08	− 13.2
0.1		91.28	54.49	− 0.02	− 11.66	− 12.95	− 13.08	− 13.01
0.2		91.69	57.96	1.43	− 11.49	− 12.95	− 13.07	− 12.78
0.3		92.08	61.75	3.25	− 11.28	− 12.94	− 13.06	− 12.58
0.4		92.44	65.89	5.58	− 10.99	− 12.93	− 13.05	− 12.37
0.5		92.77	70.41	8.69	− 10.6	− 12.91	− 13.03	− 11.94
0.6		93.06	75.31	13.03	− 10.01	− 12.89	− 13.00	− 11.57
0.7		93.3	80.53	19.51	− 9.04	− 12.86	− 12.98	− 11.32
0.8		93.46	85.89	30.23	− 7.16	− 12.79	− 12.95	− 11.03
0.9		93.53	90.88	51.01	− 1.83	− 12.6	− 12.92	− 10.83
1		93.41	93.63	**94.09**	91.34	38.92	− 12.9	− 10.82

## Conclusion

This study proposes an LSTM model for the prediction of suspended sediment in the Johor river in Malaysia. The prediction model was trained on the daily sediment and daily discharge data. The model was trained and validated on 80% of the data and tested on the remaining 20% of the data. Four different models were analysed for suspended sediment prediction, such as ElasticNet Linear Regression, MLP neural network, Extreme Gradient Boosting and Long Short-Term Memory. These models were trained on four different scenarios: daily, weekly, 10-daily, and monthly. This study was divided into three experiments. The first experiment was for the development of the LSTM model for one day ahead prediction of suspended sediments. The results of experiment one showed that LSTM outperformed other models with the regression values as 92.01%, 96.56%, 96.71%, and 99.45% in daily, weekly, 10-days, and monthly scenarios, respectively. The second experiment was for sensitivity analysis of the inputs. The second experiment results showed that the LSTM model performs best when discharge at time t is used as an input for predicting sediment at time t, with regression values obtained as 84.24%. 96.52%, 96.48%, and 98.6% for daily, weekly, 10-days, and monthly scenarios, respectively. The third experiment compared LSTM and ElasticNet LR (with and without normalization) for four scenarios in terms of regression values for one to seven-time steps ahead. The third experiment's outcome was that the LSTM model has outstanding performance in predicting the sediment for one day and seven days ahead. In summary, using LSTM has improved the evaluation metrics by obtaining an increase in the coefficient of determination R^2^ and a decrease in RAE, RSE, RMSE, and MAE. This study's limitation is related to the size of data collected for the period of 1988 to 1998. Collecting more training data can improve data-hungry models of deep learning by learning new patterns from new samples. Therefore, we intend to enhance the future results by retraining the proposed LSTM with future collected data. Furthermore, a combination of 1D convolutional layers with LSTM can be investigated in future work to combine both spatial and temporal features to enhance the prediction.

## Data Availability

The data that support the findings of this study are available Department of Environment Malaysia (DOE).
